# Bayesian Spatial Survival Analysis of Duration to Cure among New Smear-Positive Pulmonary Tuberculosis (PTB) Patients in Iran, during 2011–2018

**DOI:** 10.3390/ijerph18010054

**Published:** 2020-12-23

**Authors:** Eisa Nazar, Hossein Baghishani, Hassan Doosti, Vahid Ghavami, Ehsan Aryan, Mahshid Nasehi, Saeid Sharafi, Habibollah Esmaily, Jamshid Yazdani Charati

**Affiliations:** 1 Department of Biostatistics, Faculty of Health, Mashhad University of Medical Sciences, Mashhad 913767-3119, Iran; nazari951@mums.ac.ir; 2 Department of Statistics, Faculty of Mathematical Sciences, Shahrood University of Technology, Shahrood 316-3619995161, Iran; hbaghishani@shahroodut.ac.ir; 3 Department of Mathematics and Statistics, Macquarie University, Sydney, NSW 2109, Australia; hassan.doosti@mq.edu.au; 4 Social Determinants of Health Research Center, Mashhad University of Medical Sciences, Mashhad 913767-3119, Iran; GhavamiV@mums.ac.ir; 5 Antimicrobial Resistance Research Center, Mashhad University of Medical Sciences, Mashhad 917669-9199, Iran; AryanE@mums.ac.ir; 6 Centre for Communicable Diseases Control, Ministry of Health and Medical Education, Tehran 141994-3471, Iran; mnasehi@yahoo.com (M.N.); sharafisaeed@yahoo.com (S.S.); 7 Department of Biostatistics, Health Sciences Research Center, Addiction Institute, Mazandaran University of Medical Sciences, Sari 484711-6548, Iran

**Keywords:** pulmonary tuberculosis, duration to cure, altitude, spatial survival models, interval censored, Iran

## Abstract

*Mycobacterium tuberculosis* is the causative agent of tuberculosis (TB), and pulmonary TB is the most prevalent form of the disease worldwide. One of the most concrete actions to ensure an effective TB control program is monitoring TB treatment outcomes, particularly duration to cure; but, there is no strong evidence in this respect. Thus, the primary aim of this study was to examine the possible spatial variations of duration to cure and its associated factors in Iran using the Bayesian spatial survival model. All new smear-positive PTB patients have diagnosed from March 2011 to March 2018 were included in the study. Out of 34,744 patients, 27,752 (79.90%) patients cured and 6992 (20.10%) cases were censored. For inferential purposes, the Markov chain Monte Carlo algorithms are applied in a Bayesian framework. According to the Bayesian estimates of the regression parameters in the proposed model, a Bayesian spatial log-logistic model, the variables gender (male vs. female, TR = 1.09), altitude (>750 m vs. ≤750 m, TR = 1.05), bacilli density in initial smear (3+ and 2+ vs. 1–9 Basil & 1+, TR = 1.09 and TR = 1.02, respectively), delayed diagnosis (>3 months vs. <1 month, TR = 1.02), nationality (Iranian vs. other, TR = 1.02), and location (urban vs. rural, TR = 1.02) had a significant influence on prolonging the duration to cure. Indeed, pretreatment weight (TR = 0.99) was substantially associated with shorter duration to cure. In summary, the spatial log-logistic model with convolution prior represented a better performance to analyze the duration to cure of PTB patients. Also, our results provide valuable information on critical determinants of duration to cure. Prolonged duration to cure was observed in provinces with low TB incidence and high average altitude as well. Accordingly, it is essential to pay a special attention to such provinces and monitor them carefully to reduce the duration to cure while maintaining a focus on high-risk provinces in terms of TB prevalence.

## 1. Introduction

Tuberculosis (TB) remains a significant reason for a public health problem and is the leading cause of a single infectious agent, ranking above HIV/AIDS. This disease is preventable and curable if treated in time [1,2,3]. Despite the progress in recent decade in the effective treatment of TB, the disease is still regarded as a primary global public health challenge and tops the rankings of infectious diseases [4]. TB is found in two types in humans, including pulmonary tuberculosis (PTB) and extrapulmonary tuberculosis (EXTB). Generally, 80% of TB cases are PTB, which has two types: smear-positive and smear-negative. Since untreated people with smear-positive PTB can infect 10 to 15 healthy people annually, these cases are the primary reason of microbial transmission in the community [5,6,7]. Globally, all bacteriologically confirmed cases, including smear-positive and smear-negative PTB, should appropriately be treated. However, it is difficult to confirm smear-negative PTB cases in most laboratories in Iran due to the lack of mycobacterial culture facilities. Therefore, according to the Iranian national guidelines [8] in accordance with the WHO guidelines [9,10], smear-positive cases are usually the priority of treatment. In this way, timely and effective treatment of more infectious cases can profoundly impact the control of the disease. In 2014, in line with sustainable development goals, the WHO settled the “End TB Strategy” to decline TB deaths by 95.0% and TB incidence by 90.0% relative to 2015 until 2035 [11,12]. However, there are obstacles to achieving this goal; for example, it requires new and sustainable diagnostic tools, drugs that shorten the duration of treatment, and improved quality of care [13]. 

Based on some reports of the Iranian Ministry of Health and Medical Education (MOHME), the incidence of TB in the country has declined from 23 cases per 100,000 inhabitants in 2000 to 14 cases per 100,000 inhabitants in 2018 [14,15]. Despite this significant decline in the incidence of TB and the TB mortality rate over the last two decades in Iran, this disease still affects the economy and the health system. 

WHO recommended the Directly Observed Treatment Short-Course (DOTS) strategy, which aims at detecting 70% of infectious cases and curing 85% of them [16,17]. Anti-TB treatment duration is at least six months for new patients, or longer if there is resistance [18]. Based on the standard guidelines, standardized TB treatment is divided into two phases: the intensive phase (IP) for two months and the continuation phase (CP) for four months [19], but, according to an Asia-Pacific study, clinicians may decide to treat patients for a longer time if a TB patient is immunocompromised (e.g., HIV, diabetes mellitus), and exhibits vast lung contention like lung cavities at the start of the treatment. In this case, in both IP and CP phases of treatment, if the clinicians decide to extend the treatment period by ≥2 weeks, it is considered as a prolonged treatment phase [20]. Prolonged treatment has a noticeable burden on patients and health system and enhances the risk of non-adherence, non-completion treatment, and unfavorable TB treatment outcome [21,22]. Poor adherence to TB treatment, which is an outcome of a long treatment course, may prolong infectiousness and enhance the risk of drug resistance, relapse, and death, as well as onward transmission [1,23]. Therefore, in order to prevent and reduce the mentioned problems, it seems necessary to evaluate the duration to cure (the time interval between the initiation and end of treatment) and its determinants in smear-positive PTB patients. Furthermore, since TB is a socio-economic disease, investigating the patterns of duration to cure in Iranian patients is essential due to differences in culture, lifestyle, genetics, and diets of different ethnic groups, as well as climatic diversity in the country. As far as the researchers investigated, in spite of the evidence of a strong association between prolonged duration to cure and mentioned problems such as poor adherence, non-completion treatment, unfavorable TB treatment outcome, drug resistance, relapse, etc., few studies have been conducted in this regard. 

The analysis of survival data in which the outcome variable is time until an event of interest occurs, has many applications in medicine, public health, etc. A key feature of this type of data is the presence of censored observations [24,25]. In most public health studies, survival data are gathered over distinct spatial locations. Mostly, the adjacent locations may be more similar than those from distant regions due to alike common risk factors of disease such as air pollution, access to healthcare, altitude, and mean family income. Thus, there are disparities in survival rates between various regions occasionally. In other words, there is the potential for survival time to vary among locations. In such circumstances, conventional survival models do not capture spatial structures in the model and an alternative approach is to use the spatial survival model. In these models, spatial variation is modeled as a spatial random effect or spatial frailty to represent different clusters or geographical regions [26]. Many studies have been proposed concerning the survival model with spatial components [27,28,29]. It is explicit that spatial referencing is crucial for comprehending geographical variations in the disease outcome. 

Recently, machine learning models have been used in various areas of TB, including predicting the treatment outcome of TB patients [30,31,32], adherence research [33] and diagnostic algorithm for active TB (ATB) [34]. A study by Atif et al. investigated the durations of the IP and CP phases of TB treatment and their predictors among new smear-positive PTB patients in Malaysia [20]. A study by Seyedagha et al. examined the effect of geographical patterns on the improvement of patients with PTB based on a log-logistic model in Iran [35]. Their findings highlighted that cure duration among PTB patients significantly varies by location. However, they implemented only the log-logistic model with one form of spatial prior (a CAR prior). They also did not compare the fitness of several parametric models with different spatial priors. Further, they did not consider the main variables such as pretreatment weight, delayed diagnosis, etc. in the model. A study by Dangisso et al. indicated that TB case notification rates (CNRs) and treatment success are inversely associated with altitude [36]. Also, numerous studies reported the relationships between altitude and TB incidence [37,38,39,40]. Although previous studies have emphasized the effect of altitude therapy on TB disease, there is no evidence about the association between altitude and duration to cure of PTB patients [41]. 

As mentioned above, many of the studies investigated the relationships between the mentioned risk factors and the TB incidence. In contrast, a few studies have evaluated the impacts of these factors on the duration to cure of smear-positive PTB patients. Accordingly, more studies are needed on this subject. Suppose areas with long cure time are evidenced in reports. In that case, policymakers as an essential professional can act in the development of actions to prevent the unfavorable TB treatment outcome and control TB in these places. To address this issue, the main purpose of this study was to investigate the possible geographical variation of duration to cure and its associated factors among new smear-positive PTB patients in Iran by the Bayesian spatial survival model.

## 2. Material and Methods

### 2.1. Design 

We executed a national historical cohort study in Iran from March 2011 to March 2018 based on data from the National Tuberculosis and Leprosy Registration Center of Iran’s MOHME. During the study period, a total of 40,151 smear-positive PTB cases were diagnosed in Iran. These cases had been diagnosed by 61 Iranian medical universities from March 2011 to March 2018, covering all 429 counties of Iran’s 31 provinces. 

### 2.2. Study Participant and Procedure

The study’s inclusion criteria was a new smear-positive PTB case with high transmission power in the community and priority of treatment. So, 35,608 (88.70%) new cases were included in the study. Meanwhile, the exclusion criteria of the study were (a) recorded as a misdiagnosed TB, and (b) missing values in the covariates of interest or survival time (duration to cure). Consequently, 346 misdiagnosed TB cases and 518 cases with missing values were removed from the data. Finally, the total of 34,744 new smear-positive PTB cases were included in the analysis. In addition to examining acid-fast bacilli (AFB) smear-positive sputum at the start of treatment, according to the national instructions and WHO guidelines, these new cases are also monitored at the end of months 2, 3 (if the specimen obtained at the end of month 2, sputum smear microscopy should be obtained at the end of month 3), 4, and 6 via microscopic observation of the sputum. Moreover, it should be noted that for each patient at the beginning of treatment and in the follow-up stages, if the results of the reference laboratory smear were available, they replaced the results of the local laboratory smear.

The Tuberculosis and Leprosy Control Office of Iran’s MOHME uses the unique computerized questionnaire to perform registering, analyzing, and controlling TB morbidity, mortality, and its related risk factors at the national level for all 429 counties in 31 provinces of Iran. After obtaining the information registered in the TB registration uniform portal, we extracted all the demographic characteristics and other related risk factors that had a lower rate of missing values. These factors included age, pretreatment weight, sex, delayed diagnosis (the time interval between the onset of TB symptoms and diagnosis), location (urban vs. rural), bacilli density in the initial smear (1–9 bacilli determined as 1–9 AFB Per 100 immersion fields, 1+ determined as 10-99 AFB per 100 immersion fields, 2+ is determined as 1–10 AFB per 1 immersion fields, 3+ is determined as >10 AFB per 1 immersion fields), nationality (Iranian vs. other), prison condition (yes vs. no), treatment outcomes of new PTB patients based on WHO definition (cured, completed treatment, treatment failed, died, transfer out, defaulted or interrupted), and duration to cure (the time interval between the initiation of treatment and end of treatment) [42]. In the second step, the altitude from sea level of every Iran County was extracted by GIS tools.

### 2.3. Survival Time Description

Each subject’s survival time was calculated in months based on the time interval between treatment initiation and the end of treatment (duration to cure). The cured outcome is defined as an event of interest in which we encountered two types of censorship: a: Patients’ improvement assessment index is the measurement of their sputum smear measured according to a specific program and periodically at the end of months 2, 3, 4, and 6 for new patients. In reality, we only know that the patient’s cure occurred within a known period (the interval between two sputum cultures) but the exact time of cure is unknown. Such data are interval-censored data in ($L_{i}$, $R_{i};i=1,2,\ldots ,\;34744$), and we considered the last positive appointment $L_{i}$ (smear-positive result) and end of treatment $R_{i}\;\left(\mathrm{the}\;\mathrm{last}\;\mathrm{negative}\;\mathrm{result}\;\mathrm{for}\;\mathrm{cured}\;\mathrm{patients}\right).$ b: We faced with right-censored data for patients who left the treatment in the study period and were not cured. In this case, the observations are right-censored at the end of treatment. When the survival time was right censored, $L_{i}$ = End of treatment time and $R_{i}=$ extremely large, whereas for exact observations it was $L_{i}$ = $R_{i}$ [43].

### 2.4. Statistical analysis

The continuous and qualitative variables are summarized as mean ± SD or median (Q1–Q3) and frequency (%), respectively. Comparison of means of quantitative variables between the two groups was carried out by independent T-Test. Moreover, the association between two qualitative variables was assessed using Chi-square and Fisher exact test.

One of the appropriate alternative approaches in the survival-time analysis is the parametric accelerated failure time (AFT) model when the proportional hazard (PH) assumption does not hold. Often, the parametric AFT model with three conventional baseline survival distributions (normal, extreme value, and logistic) is used for survival data analysis.

Let $T_{ij}$ denote the survival time (cure duration) for subject *j* in county *i*, and $x_{ij}$ display the p-dimensional vector of covariates corresponding to $T_{ij}$, where *j* = 1, 2, …,$\;n_{i}$, *i* = 1, 2, …, *n*. The parametric AFT model is defined as follows:

(1) $$\log(T_{ij})=\mu +\beta x_{ij}+\sigma \varepsilon_{ij},$$

where *β = (*$\beta_{0}$*,*$\;\beta_{1},\ldots ,\beta_{P})$ is the vector of unknown regression coefficients, $\varepsilon_{ij}$’s are independent random errors, and *µ* and σ  are the shape and scale parameters, respectively. When $n_{i}$ *=* 1, the regular AFT model is achieved [27]. 

Findings from the study conducted by Seyedagha et al. revealed that there were remarkable spatial variations in the duration to cure of smear-positive PTB patients in Iran [35]. Furthermore, examining the Kaplan-Mayer diagram by provinces, we realized that the survival rate changes considerably with the location (not shown here). Therefore, adding spatial effects in the regular parametric AFT model improves the estimates of risk effects. A parametric AFT spatial model can be expressed as follows:

(2) $$\log(T_{ij})=\mu +\beta x_{ij}+W_{i}+\sigma \varepsilon_{ij},$$

where $\;W_{i}$’s are spatial random effects (in this study, we considered province-specific random effects, *I* = 1, 2, …, 31).

In this paper, in dealing with the possible spatial correlation of adjacent provinces, we considered three different approaches:

(a) $W_{i}$ is added to the survival model as non-spatial frailty (spatially uncorrelated random effects), and it follows an independent normal distribution.
(b) We consider a conditional autoregressive (CAR) model for spatial random effects, which was proposed by Besag et al. [44] as follows:

Let W = ($W_{1}$, $\;W_{2}$, …, $W_{n}$) denote a vector of spatial random effects (spatial frailties), where $W_{i}$ indicates the province-specific spatial frailty for province i. In the CAR model, spatial correlation between the random effects is defined via a binary n × n adjacency matrix U, with elements $U_{ik}$ composed of zeros and ones, where $U_{ik}$ = 1 if and only if regions *i* and *k* are defined to be neighbors, and is zero otherwise. For the identifiability of these models, it is usual to suppose that $\sum_{i}W_{i}$ = 0. Then, the CAR model are given by:

(3) $$W_{i}׀(U,v_{s}^{2})\sim \mathrm{Normal}({\bar{W}}_{i},\sigma_{i}^{2}),\;{\bar{W}}_{i}\;=\;\frac{\sum_{k}W_{k}U_{ik}}{U_{i+}},\;\sigma_{i}^{2}\;=\frac{v_{s}^{2}}{U_{i+}},$$

where ${\bar{W}}_{i}\;is\;$ the average of the random effects in neighboring areas, $\;U_{i+}=$ $\sum_{k}U_{ik}$ is the number of areas that share boundaries with the *ith* province, and variance weighted by the inverse of the total number of neighbors. In the presence of strong spatial correlation, this conditional variance reflects the fact that the more neighbors an area has the more information there is from its neighbors about the value of its random effect. Also, the variance parameter $v_{s}^{2}\;$ controls the amount of variation between the random effects.

(c) We also considered the convolution prior model for $W_{i}$ that was first introduced by Besag et al. [44]. This model incorporates both spatially correlated and uncorrelated random effects in order to consider the spatial correlation between clusters and the correlation of observations within clusters. A purely spatial structure of random effects can be considered as follows:
  (4) $$W_{i}=\;W_{i1}\;+W_{i2},$$

Here, $W_{i1}\;$ represents the spatial random effects introduced by a CAR prior described in the previous section. The second set of random effects $W_{i2}$ are independent random effects with zero mean and constant variance, which incorporates the correlation of observations within each cluster (province) into the model. $W_{i2}$ ~ Normal (0,$\;v_{s2}^{2}$), $v_{s2}^{2}$ is the variance parameter.

In both the regular and spatial AFT models, conventional distributions for ε are the standard normal, the standard extreme, and the logistic distributions. Hence, *T’s* survival distributions, corresponding to *ε’s* specified distributions, are the log-normal, Weibull, and log-logistic distributions, respectively [27]. Here, we examined nine different models combining three aforesaid conventional distributions for ε and three different approaches for incorporating spatial correlation in an interval-censored data setting within the Bayesian framework.

### 2.5. Bayesian Inference

Let *f*(.) and *F*(.) be the density function and cumulative distribution function (CDF) of *T,* respectively. Also, let $f_{0}\left(.\right)$ and $F_{0}\left(.\right)$ denote the density function and cumulative distribution function (CDF) of *ε*, respectively. Further, denote the survival function of *T* as *S*(.), and of *ε* as *S0*(.). We denote the observed data ($B_{ij}$, $t_{ij},\;\delta_{ij},\;x_{ij}$), where $B_{ij}=\left(t_{ijL},\;t_{ijU}\right]\;$ is the interval during which patient *j* in county *i* occurs cured outcome, $x_{ij}\;$ is the *p-dimensional* vector of covariates, and $\delta_{ij}$ is the following interval censoring indicator:

(5) $$\delta_{ij}=\{\begin{array}{cc} 1 & if\;subject\;ij\;is\;interval-censored\;\left[cured\right].\;\\ 0 & if\;censored\;at\;L_{i}=C_{i}\;\left(C_{i}\;\mathrm{being}\;\mathrm{the}\;\mathrm{censoring}\;\mathrm{time}\right). \end{array},$$

Then, given the spatial random effect, the likelihood function of the model with right, and interval-censored observations can be written as [45]:

(6) $$L(\{t_{ijL},\;t_{ijU}\}׀\;\emptyset \;,W)=\prod_{i=1}^{n}\prod_{j=1}^{n_{i}}{\left[F\left(t_{ijU}׀\;\emptyset \right)-F\left(t_{ijL}׀\;\emptyset \right)\right]}^{\delta_{ij}}*{\left[S\left(t_{ijL}׀\;\emptyset \right)\right]}^{1-\delta_{ij}},$$

where ∅ = {μ,σ,β} be the vector of parameters to be estimated. For Bayesian analysis, we consider the following prior distributions for each parameter in the model. Since there is no information about the prior distributions of the parameters, the non-informative prior distributions are selected for all parameters in this model. For each of the coefficients $\beta_{0}$, $\;\beta_{1},\ldots ,\beta_{P}$ and shape parameter *µ* in the model, we used independent vague normal priors with mean *0* and variance 1 $\times 10^{3}$. We also used a vague gamma prior for the shape parameter σ, $v_{s}^{2}$*,* $v_{s1}^{2}$*,* $v_{s2}^{2}$. 

Estimating the parameters of spatial AFT model is performed by approximating the posterior distribution of the model based on a Markov Chain Monte Carlo (MCMC) simulation algorithm in a Bayesian framework. If we employ *p(*∅) and *p*($v_{s}$) for denoting the prior distribution of ∅ and variance of the spatial random effects, respectively, then the posterior distribution is given by:

(7) $$p(\emptyset ,W,v_{s}׀\left\{t_{ijL},\;t_{ijU}\}\right)\propto L\left(\{t_{ijL},\;t_{ijU}\}׀\;\emptyset ,W\right)\;p(W׀\;v_{s})\;p(\emptyset )\;p(v_{s}).,$$

We implemented our models in OpenBUGS by employing a “zero trick”. All models were run for 75,000 iterations, with the first 35,000 iterations discarded as burn-in, and the next 40,000 samples used as the posterior analysis. The deviance information criterion (DIC) was used to compare and choose the best-fitting model [46]. Lower values of DIC indicate a better-fitting model. Furthermore, trace plots were used to assess convergence. Zoning of Iranian provinces was performed in GIS software based on the posterior median of spatial random effects W. It is worth noting that the natural break method in GIS was applied for generating the cut point on the map. All preliminary data analysis was carried out by SPSS (version 16, Institute Inc., Chicago, IL, USA) and R (version 3.6.2, www.r-project.org) at the significance level of 0.05. 

## 3. Results

**Figure 1:**
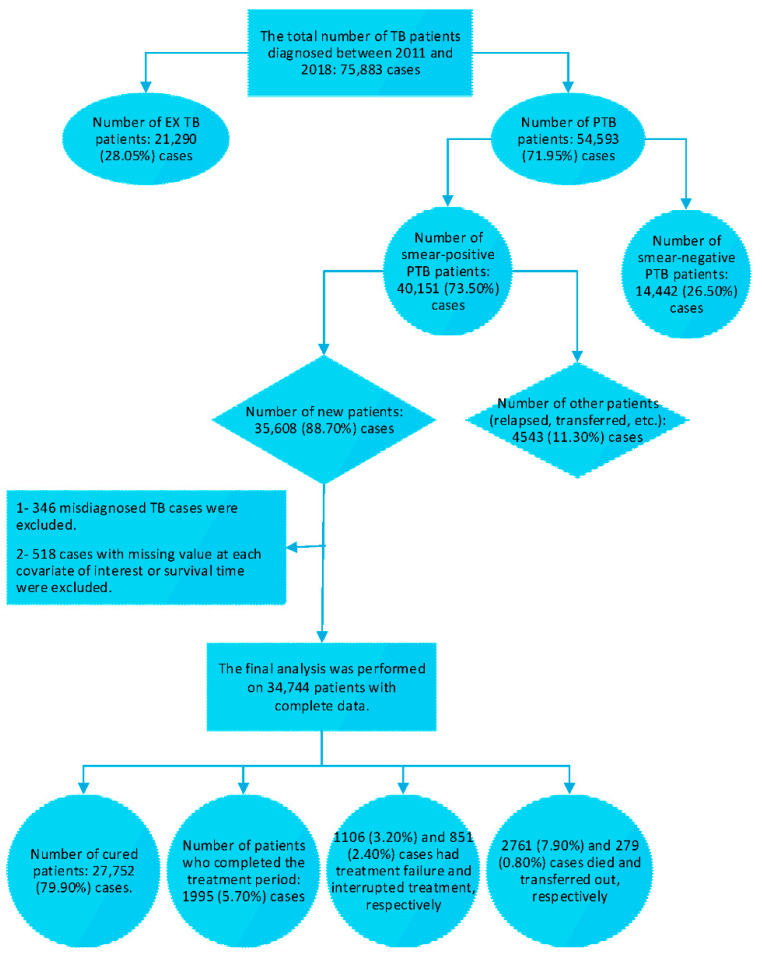
Study population. Treatment outcomes for new smear-positive PTB patients were defined according to WHO guideline [42].

Between March 2011 and March 2018, a total of 75,883 active TB cases (PTB and EXTB) were diagnosed in Iran, 35,608 of whom were new smear-positive PTB patients. Finally, 34,744 (97.60%) of patients who had complete records on all covariates of interest and survival time, were included in the study (Figure 1). 

Of the 34,744 patients, 27,752 (79.90%) experienced cured outcome as an event of interest, and 6992 (20.10%) patients were censored (1995 (5.70%) cases had completed the treatment period, 1106 (3.20%) cases experienced treatment failure, 851 (2.4%) cases had interrupted the treatment or loss to follow-up, 2761 (7.90%) cases died, and 279 (0.80%) patients transferred out). The demographic and clinical characteristics of new smear-positive PTB patients with outcome status are shown in Table 1. The mean age at diagnosis of patients was 51.26 ± 21.47 years and most patients were in the 34–63 years age group. Among the data, 18,680 (53.80%) cases were males and 16,064 (46.20%) were females. The median altitude of counties in Iran was 1027 (133–1302) meters. Gilan (Northern Iran) and Chaharmahal-Bakhtiari (western Iran) provinces with the median of 3 (1–27) and 2061 (1722–2224) meters had the lowest and highest altitude, respectively. Among patients, 15,174 (43.7%) cases had delayed diagnosis between 1 and 3 months. The median duration to cure of new smear-positive PTB patients was 6.33 (7.20–6.31) months in Iran.

In Table 2, the DIC values are given for choosing the best fitting model. The results revealed that the convolution prior with the logistic baseline (log-logistic model with convolution prior) had the smallest DIC value (24,810) among these models; so, it was chosen as the best fit model. Therefore, we conclude that there were considerable regional variations in the survival probability of new smear-positive PTB patients in Iran.

Consequently, the estimated parameters (the posterior means and 95% credible intervals) for the log-logistic model with convolution prior, as the best-selected model with the lowest DIC value, are shown in Table 3. Our results indicated that the variables sex (male vs. female), altitude (≤750 m vs. >750 m), bacilli density in initial smear (2+ vs. 1–9 Basil & 1+ and 3+ vs. 1–9 Basil & 1+), delayed diagnosis (>3 months vs. <1 months), nationality (Iranian vs. other), and location (urban vs. rural) had a significant influence on prolonging the duration to cure in the new smear-positive PTB patients. However, pretreatment weight was significantly associated with shorter duration to cure in these patients (as showed by * in the Table 3). After adjusting for other covariates in the model, it was observed that the duration to cure of patients living in areas with an altitude of >750 m was 1.05 times longer than the patients whose altitude of residence was ≤750 m. In other words, the duration to cure at an altitude of >750 m was 5% longer than at an altitude of ≤750 m, and cure occurred later at higher altitudes. Males versus females (with TR = 1.09) were associated with the prolonged duration to cure. The duration to cure of patients living in urban areas was 1.02 times that of patients living in rural areas. Duration to cure of patients with bacilli density in initial smear as 2+ and 3+ are 1.02 and 1.09 times compared to 1–9 Basil & 1+, respectively. Delayed diagnosis of >3 months against <1 month was associated with prolonged duration to cure, with a 2% increase in the duration to cure. Iranian patients had 1.02 times longer duration to cure compared to other ones. Also, higher pretreatment weight was associated with shorter duration to cure. But such variables as age, prison condition, and delayed diagnosis between 1 and 3 months did not significantly associate with duration to cure.

**Figure 2:**
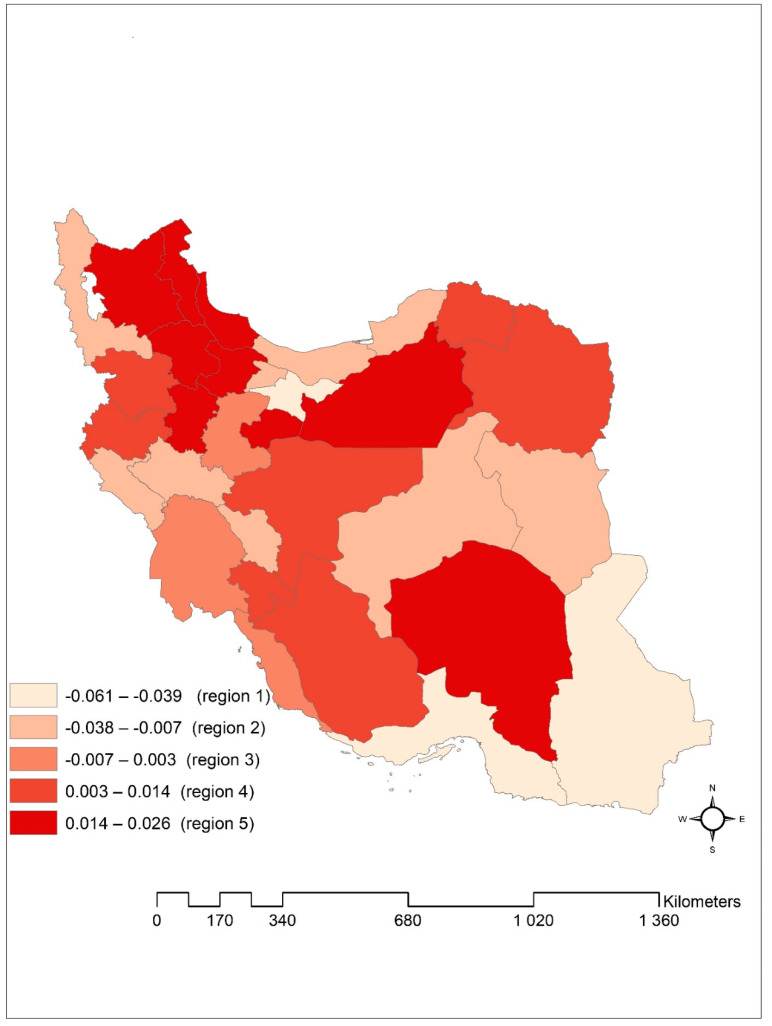
Posterior median of spatial frailties from the log-logistic model with convolution prior, Iran provinces.

To investigate the spatial patterning of duration to cure, we presented the posterior median of spatial random effects in Figure 2. The larger values of spatial random effects indicate provinces with longer duration to cure. We understood that there was a noticeable spatial variation in the duration to cure of new smear-positive PTB patients in Iran, in a way that some provinces in the north-western, middle, and eastern parts of Iran had a prolonged duration to cure. 

**Figure 3:**
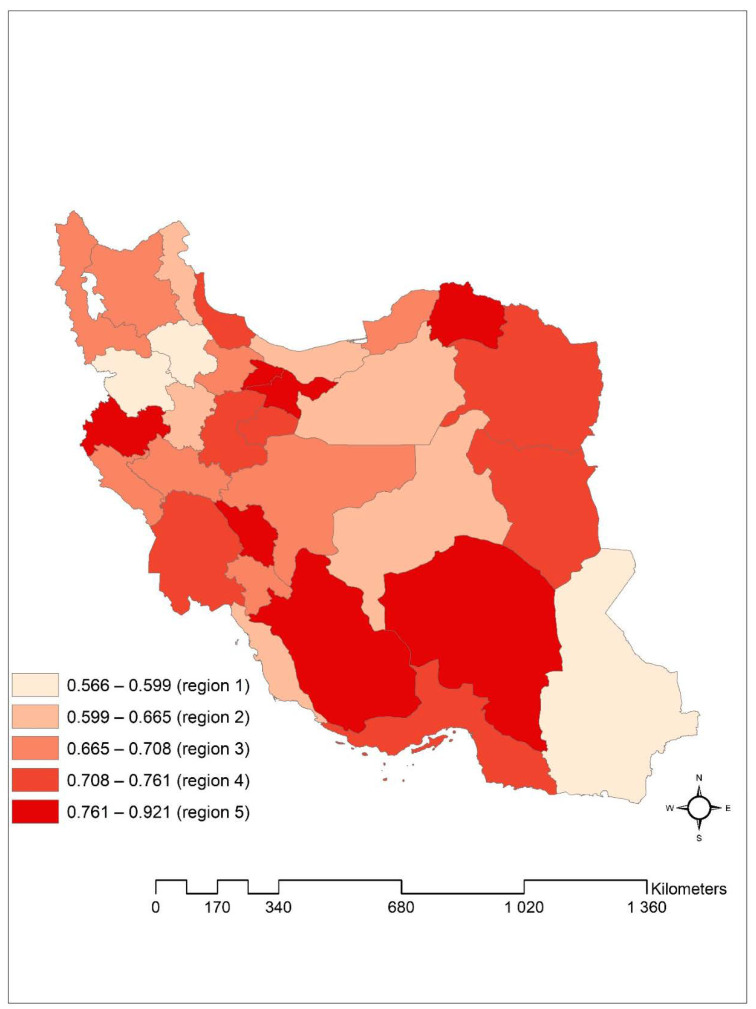
Posterior median of spatially correlated plus uncorrelated random effects from the log-logistic model with convolution prior, Iran provinces.

Figure 3 maps the posterior median of spatially correlated plus uncorrelated random effects from the best-selected model. As can be seen, there is a relatively notable geographical variation in duration to cure of these patients in Iran. 

## 4. Discussion

Due to the long treatment period of TB, the cure of these patients requires regular monitoring along with timely and continuous use of drugs. Hence, identifying the factors related to the duration to cure and its evaluation throughout the country is considered an urgent priority for controlling TB. This can substantively help in improving the treatment process and solving the existing problems in this regard. Therefore, we conducted a national population-based historical cohort study to examine the possible geographical variation in duration to cure and determining its associated factors using the Bayesian spatial survival model among new smear-positive PTB patients in Iran. In the present study, due to the nature of the data as well as the violation of the PH assumption in the Cox model, firstly we examined three common parametric AFT models with three different spatial priors including uncorrelated (non-spatial frailty), correlated (CAR prior), and both uncorrelated and correlated (convolution prior) spatial random effects. Ultimately, we concluded that the log-logistic model with convolution prior provided a better performance based on the goodness of fit criterion (DIC = 24,810), and the variance of the spatial random effect (144.60 (6.97, 977.40)) is also statistically significant. It should be noted that the estimation of regression parameters in conventional survival models can be obtained by maximizing the likelihood function; but in the spatial survival model, the posterior distribution does not have a closed form and cannot be estimated directly. Therefore, in general, the Bayesian inference framework is used, and usually, the calculation of the maximum likelihood estimates of the parameters is based on Monte Carlo numerical methods. These results demonstrate that the parametric AFT model, accounting spatial correlation compared to the model without the spatial structure, has a better performance when there is a substantive spatial variation in the data. In addition, our result regarding the consideration of spatial heterogeneity in the model by including spatial frailty is in concordance with some other studies [27,29,47]. 

The results from the Chi-square test showed that there is a significant relationship between the cured outcome with variables age groups, gender, altitude, bacilli density in the initial smear, delayed diagnosis, nationality, and location. in other words, the proportion of cured patients in females compared to males, patients <15 years vs. other age groups, people living at high altitude areas vs. people living in low altitude areas, bacilli density 2+ vs. other bacilli densities, delayed diagnosis <1 month compared to delayed diagnosis between 1 and 3 months and >3 months, other nationalities vs. Iranian, and rural vs. urban is significantly higher (Table 1). In addition, the results from our best-selected model indicated that the duration to cure of new smear-positive PTB patients had a relatively considerable spatial variation in Iran, and it is much higher in region 5 (Figure 2). If we consider the duration to cure equal to the average duration to cure of patients in this study (190 days), according to the spatial frailty values obtained from the model in the region 5, the duration to cure in this region is between exp(0.014) * 190 = 192.67 days and exp (0.026) * 190 = 195 days. This region includes Gilan, Ardabil, East Azerbaijan, Hamadan, Zanjan, Qazvin, Semnan, Qom, and Kerman provinces. These provinces are located in the northwestern, middle, and eastern parts of Iran. All these provinces except Gilan are characterized by a high average altitude of about more than 1000 m. It should also be noted that although the average altitude in Gilan province is low, most of its areas are mountainous (the Alborz mountain range is located in the west and south). In contrast, the provinces in the region 1, including Sistan-Baluchistan, Hormozgan, and Tehran had the shortest duration to cure in Iran. Similarly, if we consider the duration to cure equal to the average duration to cure of patients in this study (190 days), according to the spatial frailty values obtained from the model in the region 1, the duration to cure in this region is between exp(−0.061) × 190= 178.60 days and exp(−0.039) × 190 = 182.59 days. These results are opposite the fact that prolong durations to cure were expected in provinces with a high number of TB incidence and low average altitude because mycobacterium TB is strictly aerobic [48]. However, a longer duration to cure was observed in the provinces with a low TB incidence and high altitude. So, we found an inverse relationship between TB incidence rate and the duration to cure of PTB patients and a direct association between duration to cure and the average altitude of provinces. The possible reason could be related to close supervision, special attention, and extensive healthcare system efforts in the provinces with a high TB incidence rate. 

In addition, it was revealed that gender (male vs. female), altitude (>750 m vs. ≤750 m), bacilli density in initial smear (3+ vs. 1–9 Basil & 1+ and 2+ vs. 1–9 Basil & 1+), delayed diagnosis (>3 months against <1 month), nationality (Iranian vs. other), location (urban vs. rural), and pretreatment weight variables were significantly associated with duration to cure of new smear-positive PTB patients. To put it another way, our findings after adjusting the effect of other variables in the model presented that males had a 9% longer duration to cure compared to females. Accordingly, if the duration to cure in females is 180 days, it should be 196.20 days in males. This could be related to the weaker immune system of males, which makes them more susceptible to infectious diseases and a higher proportion of unfavorable TB treatment outcomes [49,50,51]. This result is similar to Lesourd’s [52] findings, who found a weaker immune system could postpone the duration to cure in infectious diseases. Likewise, this result may be due to the greater tendency of males to engage in high-risk behaviors such as substance and alcohol abuse, and more infection with HIV [49,53], which can lead to a weakened immune system and more severe disease progression in them [54,55]. Our result is also in line with the study performed by Seyedagha et al. [35], who reported the recovery time higher in males than females. According to our findings, the duration to cure of patients living in high-altitude areas (>750 m) was 1.05 times longer than that of patients living in low-altitude areas (≤750 m). Accordingly, if the duration to cure in patients living in low-altitude areas (≤750 m) is 180 days, this duration should be 189 days in patients living in high-altitude areas (>750 m). Time ratio (TR) is interpreted similarly for other variables in the model. To compare the results, we found no previous studies about the effect of altitude on duration to cure. Also, this might be due to the low TB incidence [36,37], low case notification rates (CNRs) of TB [36,39,40,56], lower prevalence of latent TB [57,58], and poor access to roads and health facilities in highlands [36]. In addition, the following two arguments may justify our new findings. First, some studies demonstrated that due to the strictly aerobic nature of mycobacterium TB [48], its growth, proliferation, and survival rate declines with increasing altitude and consequently oxygen restriction [59,60,61]. On the other hand, it has been proven that growing and actively proliferating bacteria are more sensitive to antibiotics than those with slow growth and metabolism [48,62]. Therefore, this issue may explain the direct relationship between duration to cure and altitude. However, further studies are required to confirm the role of altitude in the duration to cure of PTB patients. Second, according to Hui et al. [63], the pharmacokinetics of drugs changes at high altitudes, so that in order to maintain the effect of the drug for a certain period of time, it is necessary to modify the dose of the treatment regimen in the highlands. In addition, it was revealed that pretreatment weight was inversely associated with duration to cure. In this regard, Atif et al. reported that being underweight had a significant impact on prolonging the intensive phase of treatment [20]. As such, some studies revealed that underweight or below normal body mass index (BMI) is a crucial risk factor for infection [64] as well as a significant indicator of malnutrition, which may endanger the immune system [65] and prolong duration to cure in infectious diseases [52]; our findings confirm this issue. Further, we found that a higher bacilli density in the patient’s initial smear results in a longer duration to cure. Although we could not find any previous study to support this issue, it might be explained by the fact that a higher smear grade has a higher bacterial load and it has an inverse impact on the TB treatment outcome; it also prolongs the time to smear conversion [35,66,67]. Besides, the high load of bacilli causes more tissue destruction in patients due to the immune system’s weakness in controlling the disease. Moreover, antibiotics have restrictions on accessing organisms [68,69]. Our result, in this respect, is consistent with those reported in the mentioned studies. Likewise, the results of this study showed that there is a significant direct relationship between the time to cure and delayed diagnosis, and the duration to cure of patients had delayed diagnosis of >3 months compared with delayed diagnosis of <1 month, which is significantly longer by 2%. However, there is no significant difference among the duration to cure of patients with a delayed diagnosis between 1 and 3 months against <1 month. The possible explanation for this finding might be that delay in diagnosis and treatment of TB could lead to disease progression and worsening, prolong the period of infectivity, and increase the risk of poor treatment outcomes such as death, failure, and drug resistance [70,71,72]. Our findings also indicated that the duration to cure of Iranian patients was 1.02 times longer than other ones. In our study, 96.50% of foreign nationals were Afghan refugees, who had a noticeable impact on TB transmission in Iran [73] because Afghanistan is the eastern neighbor of Iran and it is known as a country with a high TB burden [74]. Also, most Afghan refugees migrate to eastern provinces of Iran, including Sistan-Baluchistan and Khorasan Razavi, which have the highest incidence of TB in Iran [15,73]. Besides, a study in Iran showed that Afghan patients with PTB were younger and had more severe disease [75] compared to Iranian PTB patients. Therefore, the lower duration to cure observed in our study among other patients might be due to the close supervision, specific attention, and appropriate performance given by Iranian health policymakers for controlling TB in these high-risk patients and provinces. It is also notable that the Iranian government provides health and treatment services including free TB treatment to all foreign nationals, regardless of their nationality, which has led to an increase in the number of reports of some diseases among immigrants, particularly of Afghan refugees, in Iran [15,76]. This study also reported a significant relationship between duration to cure and place of residency, and the duration to cure of urban patients is 1.02 times longer than rural patients. A study by Dangisso et al. in Ethiopia revealed that the active case-finding intervention in rural areas has a significant contribution in increasing the TB CNRs and can also increase the level of access to TB care centers in rural communities as well awareness about TB and help in the timely diagnosis and treatment of this disease [36]. Another reason for the difference in duration to cure between rural and urban patients may be due to the fact that hygienic conditions are less observed in rural life than urban life. As a result, rural patients are more exposed to various microbial and infectious factors from childhood, which strengthens and trains their immune system [77,78,79,80]; this can prolong duration to cure in the urban patients [52].

Based on the results of our study, there was no significant relationship between prison condition and duration to cure. This is inconsistent with the study’s findings by Seyedagha et al. in Iran [35]. The reason for this incompatibility may be related to considering more variables as well as modeling the data with different approaches. Although the time to cure was prolonged with increased age in our study, the relationship was not significant. This is compatible with the research performed by Seyedagha et al. in Iran [35] as well as a study by Horne et al. in the United States [81], in which age had no significant relationship with time to recovery and time to smear conversion, respectively. 

The main strength of this study is the use of national registry data in Iran between 2011 and 2018. As far as the researchers investigated, this is the first study that has examined different parametric AFT models with three different spatial priors simultaneously for modeling the duration to cure in PTB patients. In addition, our study attempted to consider the property of interval censorship of data in the analysis and assess the effect of average altitude on the duration to cure of PTB patients. 

However, the current study had some limitations. First, since registry-based data was used in this study, the TB patients’ information about some variables, such as HIV status; HIV risk factors including history of injecting, drug use, history of high-risk sexual behavior, etc.; TB risk factors including DM, head and neck cancer, chronic kidney disease, silicosis disease, etc.; and the history of TB were not entirely recorded and many cases were registered as unspecified. To tackle this issue, we included the frailty term in our models for accounting the unobserved heterogeneity induced by unobservable risk factors [47]. Based on our findings, incorporating the frailty term in all our models improved the performance of models. The small values of the estimated frailty effect indicate that the covariates entered to the model explain most heterogeneities. Second, the patients’ height was defined in the TB registration system from 2014 onwards and before that they were not completely registered. So, it was not possible to calculate BMI and examine its relationship with the length of treatment. 

It is suggested that future studies investigate the spatial modeling of this data using other spatial structures such as the Leroux prior one [82]. It is also recommended that spatial models that capture the spatial structure in the model without using random effects be examined. The spatiotemporal survival model could also be fitted to investigate spatiotemporal heterogeneity in these data. Moreover, to gain better insight into the relationship between altitude and duration to cure, further studies with considering climate and socioeconomic variables should be done in this field, and if our findings are corroborated, it is recommended that the altitude of the patient’s residence be recorded in the data using a GIS device. 

## 5. Conclusions

In summary, our findings revealed that the log-logistic model with convolution prior had better performance compared to other models. Moreover, it could be concluded that there is a relatively noticeable geographical variation in the duration to cure among new smear-positive PTB patients in Iran. Identifying provinces with longer duration to cure could help policymakers to design better policies while allocating resources; as a result, this could improve the provision of health care services. Last but not least, our study also found that the variables gender, altitude, bacilli density in the initial smear, delayed diagnosis, nationality, and location had a significant impact on prolonging the duration to cure, and pretreatment weight was substantively associated with shorter duration to cure in new smear-positive PTB patients in Iran. It is recommended that telemedicine potentials be used to increase patients’ adherence to treatment, especially in males. We also recommend holding retraining courses to increase the awareness of primary care physicians and early detection of TB patients, implementation of screening programs for latent TB, performing screening in the suburbs, and evaluating HIV in TB patients, especially in males. This could be the foundation for further planning to improve the treatment process, develop actions to prevent unfavorable TB treatment outcome, control TB, and also reduce duration to cure in other endemic TB regions worldwide.

## Figures and Tables

**Table 1:** Baseline characteristics of the new smear-positive PTB patients in Iran (n = 34,744) and results of single risk factor analysis.

Characteristics	Overall	Cured		*P*
		No	Yes	
Age				<0.001 * <0.001 *
Mean ± SD	51.26 ± 21.45	54.03 ± 21.75	50.56 ± 21.34	
<15 y	503 (1.40)	79 (15.70)	424 (84.30)	
15–35 y	10,317 (29.70)	1803 (17.50)	8514 (82.50)	
35–63 y	11,789 (33.90)	2373 (20.10)	94.16 (79.90)	
>63 y	12,135 (34.90)	2730 (22.60)	9398 (77.40)	
Weight	54.60 ± 13.09	53.49 ± 13.33	54.88 ± 13.01	<0.001 *
Gender				<0.001 *
Female	16,06 4(46.20)	2548 (15.90)	13,516 (84.10)	
Male	18,68 0(53.80)	4444 (23.80)	14,23 6(76.20)	
Altitude (meter)				
Median (Q1–Q3)	1027 (133–1302)	1062 (214–1312)	1027 (128–1264)	<0.001 *
≤750	13,692 (39.40)	2275 (16.60)	11,417 (83.40)	<0.001 *
>750	21,052 (60.60)	4717 (22.40)	16,335 (77.60)	
Bacilli density in initial smear				<0.001 *
1–9 Basil	2226 (6.40)	439 (19.70)	1787 (80.30)	
1+	11,759 (33.80)	2217 (18.90)	954 2(81.10)	
2+	7786 (22.40)	1453 (18.70)	633 3(81.30)	
3+	12,97 3(37.30)	2883 (22.20)	10,09 0(77.80)	
Delayed diagnosis (month)				<0.001 *
<1	10,935 (31.50)	2074 (19.00)	8861 (81.00)	
1–3	15,174 (43.70)	2991 (19.70)	12,183 (80.30)	
>3	8635 (24.90)	1927 (22.30)	6708 (77.70)	
Nationality				0.007 *
Other	4935 (14.20)	1063 (21.50)	3872 (78.50)	
Iranian	29,809 (85.80)	5929 (19.90)	23,88 0(80.10)	
Prison condition				0.42
No	32,93 1(94.80)	6614 (20.10)	26,317 (79.90)	
Yes	1813 (5.20)	378 (20.80)	1435 (79.20)	
Location				<0.001 *
Rural	11,578 (33.30)	2027 (17.50)	955 1(82.50)	
Urban	23,166 (66.70)	4965 (21.40)	18,20 1(78.60)	

* Significant at level of 0.05, Values are reported as frequency (percent), mean ± SD or median (Q1–Q3).

**Table 2:** A comparison of goodness-of-fit (DIC values) between the three cases of the AFT spatial models.

Model	Independent	CAR Prior	Convolution Prior
		DIC	
Log logistic	25,110	25,080	24,810
Weibull	30,000	29,970	29,890
Log normal	26,350	26,330	26,290

**Table 3:** The results from the best fit AFT spatial model (Spatial log-logistic model with Convolution Prior).

Characteristics (Ref)	Mean (SD)	(95% CI)	Time Ratio
Age	0.00009 (0.0001)	(−0.0002, 0.0004)	1.00009
Weight	−0.002 (0.0003)	(−0.003, −0.001)	0.998 *
Gender (female)			
male	0.09 (0.007)	(0.07, 0.11)	1.09 *
Altitude (≤750)			
>750	0.05 (0.015)	(0.02, 0.08)	1.05 *
bacilli density in initial smear (1–9 Basil & 1+)			
2+	0.02 (0.009)	(0.008, 0.04)	1.02 *
3+	0.09 (0.007)	(0.07, 0.10)	1.09 *
Delayed diagnosis (<1 month)			
1–3	0.01 (0.008)	(−0.005, 0.02)	1.01
>3	0.02 (0.009)	(0.01, 0.04)	1.02 *
Nationality (Other)			
Iranian	0.02 (0.011)	(0.001, 0.04)	1.02 *
Prison condition (No)			
Yes	−0.008 (0.015)	(−0.03, 0.02)	0.992
Location (Rural)			
Urban	0.02 (0.008)	(0.006, 0.03)	1.02 *

* The credible interval does not contain zero; Time Ratio = The exponential of each coefficient.
